# Improving the Hydrophobicity of Powder Activated Carbon to Enhance the Adsorption Kinetics of Per- and Polyfluoroalkyl Substances

**DOI:** 10.1021/acsestwater.4c01222

**Published:** 2025-04-07

**Authors:** Elliot Reid, Qingquan Ma, Lan Gan, Jiahao He, Thomas Igou, Ching-Hua Huang, Yongsheng Chen

**Affiliations:** School of Civil and Environmental Engineering, Georgia Institute of Technology, Atlanta, Georgia 30332, United States

**Keywords:** powder activated carbon (PAC), per- and polyfluoroalkyl Substances (PFAS), adsorption, drinking water treatment, density functional theory (DFT)

## Abstract

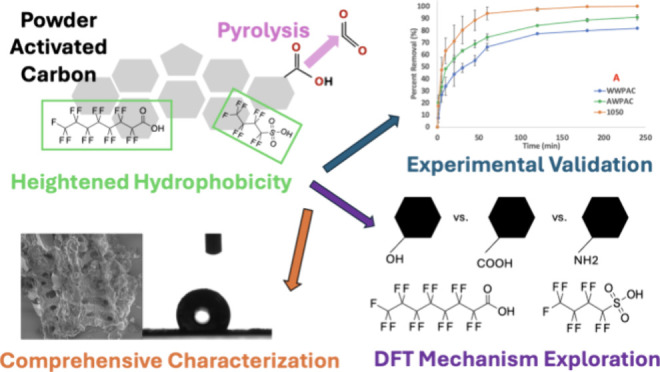

Per- and polyfluoroalkyl substances (PFAS) are difficult to treat by using conventional drinking water treatment technologies. Herein, we upgrade a commercially available powder activated carbon (PAC) via an acid wash and pyrolysis to amplify hydrophobicity and enhance PFAS adsorption. Minimal differences in overall surface area, micropore volume and area, and external surface area were observed between acid-washed and pyrolyzed PACs. X-ray photoelectron spectroscopy, contact angle measurements, and scanning electron microscopy evidenced ∼5% reduced oxygen content and noticeable hydrophobicity increases for the pyrolyzed PAC, without altering morphology. Adsorption isotherms of perfluorooctanoic acid (PFOA) showed no major increases to adsorption capacity, but more rapid adsorption kinetics of PFOA and perfluorobutanesulfonic acid (PFBS) to the pyrolyzed PAC, in both low and high PFAS concentration tests, were revealed in both reagent water and synthetic natural organic matter, with overall greater removal values (e.g., ∼90% removal vs 60%, in water after 1 h at 2 mg/L PFOA). PFOA and PFBS adsorption behavior adhered to pseudo-second-order kinetics (*R*^2^ = 0.843–0.992). Density functional theory calculations quantitatively evaluated adsorption energies of PFOA and PFBS onto a graphene skeleton containing different organic functional groups, finding supportive outcomes. This work greater informs the importance of hydrophobicity for PFAS adsorption onto PAC.

## Abstract 2

Pyrolyzed powder activated carbon demonstrates superior adsorption kinetics for several per- and polyfluoroalkyl substances, which is confirmed via density functional theory.

## 1 Introduction

Per- and polyfluoroalkyl substances (PFAS) are a class of over 3000 synthetic organic compounds with various functional group moieties that are widely used in industrial manufacturing and are components of many commonplace products such as fire retardants, nonstick cookware, and consumable packaging.^1^ The carbon–fluorine bond, one of the strongest bonds in organic compounds (105.4 kcal/mol bond dissociation energy)^2^ and a unifying feature of all PFAS, imparts notorious recalcitrance and subsequently makes their removal from water matrices particularly challenging. PFAS are ubiquitous in surface water, groundwater, wastewater treatment (WWT) plants, and drinking water treatment (DWT) plants and have been present in water sources long before society became cognizant of their potential consequences.^3−5^ A case example concerns the Cape Fear River in Wilmington, North Carolina, where, in 2017, roughly 200,000 residents discovered that they were exposed to elevated concentrations of hexafluoropropylene oxide dimer acid (HFPO-DA, or more commonly, GenX) and other PFAS found in their drinking water due to industrial discharges from an upstream manufacturing plant (potentially since the 1980s).^6^

Unfortunately, due to the sheer number of PFAS and their diverse physicochemical properties, their effects on human health and the environment are largely unknown; however, many PFAS have been linked to or are suspected to increase the risk of nefarious human health effects (e.g., pregnancy complications, alterations to epigenetic machinery),^7−12^ thus adding merit to engineering precautionary principles to protect public health.^13^ In 2016, the U.S. Environmental Protection Agency (EPA) established a Lifetime Health Advisory for combined perfluorooctanoic acid (PFOA) and perfluorooctanesulfonic acid (PFOS) at 70 ng/L,^14,15^ and in 2023, the EPA proposed the National Primary Drinking Water Regulation (NPDWR) to reduce morbidity risks associated with long-term PFAS exposure from drinking water. Specifically, this legislation aims to establish more stringent maximum contaminant level goals (MCLGs) for six PFAS: PFOA, PFOS, GenX, perfluorononanoic acid (PFNA), perfluorohexanesulfonic acid (PFHxS), and perfluorobutanesulfonic acid (PFBS). In 2024, this goal became partly successful when the EPA set enforceable MCLs for PFOA and PFOS at 4.0 ng/L, individually.^16^

Since conventional “Plus” DWT approaches (i.e., chemical dosing to remove contaminants) do little to mitigate PFAS concentrations in finished water and also create cyto- and genotoxic disinfectant byproducts (DBPs) *in situ*,^17^ many authorities will need to invest in and implement “Minus Approach” treatment technologies (i.e., use of modern physical and biological technologies to remove contaminants from solution) to remove PFAS to acceptable thresholds that comply with current and foreseeable MCLs.^18^ The high technology readiness level (TRL) platforms of granular activated carbon (GAC) and powder activated carbon (PAC) offer a realistic and cost-effective avenue to remove and sequester PFAS from water supplies. The predominant mechanisms governing PFAS adsorption onto AC are electrostatic interactions, hydrophobic interactions, and ion exchange.^19^ AC boasts high surface areas, an internal porous structure consisting of macro, meso, and micropores, and a wide spectrum of oxygenated functional groups; furthermore, it can be derived from either fossil-based or lignocellulosic precursors, offering greater sustainability potential and the advancement of circular economies.^20,21^ While AC has demonstrated itself as an effective technology to remove many PFAS, particularly the widely studied PFOA and PFOS, AC is not a “silver bullet” for PFAS remediation and suffers from various limitations: namely, lower affinities for short-chain PFAS, selective adsorption in complex water matrices, capacity saturation and breakthrough, and competing ion effects.^22−24^

Many studies have examined the effectiveness of using carbonaceous sorbents (e.g., GAC and PAC, carbon nanotubes, AC fibers and felts) to adsorb PFAS,^25,26^ with many further attempting various routes of modification to enhance precursor performance. For instance, Ilango et al. impregnated AC with goethite (α-FeOOH) to improve the overall adsorption of a PFAS mixture containing 13 compounds (234 mg/g total PFAS); the authors attribute this enhancement to both hydrophobic interactions and enhanced electrostatic interactions of PFAS head groups and iron cations.^27^ Ramos et al. modified GAC with the cationic polymer polydiallyldimethylammounium chloride (polyDADMAC) to improve the adsorption of several PFAS, notably PFBA (167.5 mg/g), even in the presence of competing ions, suggesting favorable electrostatic interactions between PFAS head groups and cationic polyDADMAC.^28^ Liu et al. additionally worked with polyDADMAC-stabilized PAC in fixed-bed column tests, finding increased PFOA and PFOS retention by 3 orders of magnitude compared to control columns.^29^ Besides carbonaceous materials, nanomaterials such as metal–organic-frameworks (MOFs) and covalent-organic-frameworks (COFs) have demonstrated high PFAS adsorption capacities (e.g., 300–700 mg/g PFOA); however, these currently are far from the technology readiness levels (TRLs) and cost-effectiveness necessary for large-scale implementation.^30^

It is hypothesized that by improving a carbonaceous sorbent’s hydrophobicity or its overall positive surface charge (i.e., inducing basic functional groups), greater PFAS adsorption performance metrics can be obtained since many PFAS targets are hydrophobic and are negatively charged at typical environmental water pH ranges.^31^ The naturally occurring acidic character of AC is typically governed by hydrophilic oxygen-containing surface groups (e.g., carboxylic, lactone, quinone, etc.).^32^ Reducing these oxygenated moieties theoretically increases the hydrophobicity. Thermal pyrolysis has been shown as one route to effectively achieve this goal,^33−35^ and pyrolysis is widely used in various preparation pathways for carbonaceous materials (e.g., pyrolysis of biomass for biochar production).^36^ Regarding enhancements to surface basicity, numerous studies have examined various pathways to nitrogenate carbonaceous materials and bolster positive surface charge since basic sites rich in π electrons are important for PFAS adsorption.^37,38^ Such methods include ammonia gas pyrolysis and amine functionalization via multistep synthesis pathways.^39−42^ For example, Zhi and Liu found that exposing various GAC materials to high-temperature in the presence of ammonia gas formed numerous basic, N-containing groups on the surface, which improved overall PFOA and PFOS adsorption capacity compared to the precursor materials.^43^

In this work, we investigate both (1) hydrochloric acid (HCl) treatment of a commercially available PAC and (2) thermal pyrolysis of this acid-treated PAC to determine how increased hydrophobicity can bolster PFAS adsorption performance metrics. First, we perform a comprehensive characterization suite of the adsorbent surface chemistry and properties. Then, we validate performance enhancements by (A) developing adsorption isotherms for PFOA to see if adsorption capacity can be increased, (B) kinetic examinations of PFOA and PFBS removal in both ultrapure water and in a synthetic NOM solution (to test the effect of a long-chain and short-chain PFAS with different functionality on performance), and (C) a mixed PFAS removal test employing the six aforementioned PFAS on the EPA’s list of concern. Finally, we conduct density functional theory (DFT) calculations to examine the adsorption energies of PFOA and PFBS interacting with a graphene skeleton containing various oxygen- and nitrogen-containing functional groups to validate mechanisms of enhancement. Insights gained from this research can guide future optimizations to enhance PFAS treatment and inspire the design of improved carbonaceous sorbents.

## 2 Materials and Methods

### 2.1 Chemical Reagents and Materials

All chemicals utilized were purchased from Sigma-Aldrich (Darmstadt, Germany) with the exception of GenX, which was purchased from TRC Chemicals (Toronto, Canada). Commercially available PAC derived from coconut shell (G.C. Powdered S, sieved at 100 mesh (99 min), 200 mesh (95 min), and 325 mesh (90 min)) was purchased from the General Carbon Corporation (Paterson, N.J.). Humic acid sodium salt was used to create a synthetic NOM solution. Reagent-grade deionized (DI) water with a resistivity of 18.2 MΩcm^–1^ and total organic carbon (TOC) concentrations ranging from 5 to 25 parts-per-billion (ppb) was produced by a water purification system (Milli-Q Benchtop Lab Water Purification System, Millipore Sigma, Darmstadt, Germany).

### 2.2 PAC Preparation

Approximately 20 g of the precursor PAC material was washed in ample DI water overnight and was filtered the next day using filter flask vacuum filtration. This PAC, called water-washed PAC (WWPAC), was then dried overnight at 105 °C and stored in a desiccator until further use. A similar procedure was used to create acid-washed PAC (AWPAC); roughly 20 g of the commercial PAC was washed with 1 M HCl overnight and subsequently vacuum filtered, washed with DI water, and allowed to dry at 105 °C overnight. To test the effect of pyrolysis on the PAC, 5 g of AWPAC was placed into a ceramic boat in a tube furnace and was pyrolyzed under argon flow (∼0.1 ft^3^/h) for ∼3 h at 1050 °C. Heating ramps were uncontrolled. After the PAC cooled to ∼50 °C, the argon flow was turned off and the PAC was removed from the tube furnace; the resultant material is referred to here as PAC-1050.

### 2.3 PAC Characterization

The surface chemistry and physical properties of the various PAC materials were characterized by using several techniques. Scanning electron microscopy (SEM) was performed using a Hitatchi SU8010 under 1.0 kV using deceleration mode at a 6.6 mm working distance; the PAC samples were dispersed in water and placed onto copper tape and vacuum-dried prior to examination. X-ray photoelectron spectroscopy (XPS) was performed using a Thermo Scientific K-Alpha XPS (Waltham, M.A.) to examine elemental composition; the PAC samples were held under vacuum at 25 in.Hg overnight prior to analysis. All spectra were referenced to the C 1s peak at 284.8 eV, and peak fitting was conducted via Avantage. Contact angle measurements were performed using a goniometer/tensiometer (Ramé-hart, Model 250) with a leveled sample holder; the powder samples were captured and hand-compressed into a thin layer on top of a glass microscope to create a “plate” that was analyzed via the sessile drop method.^44^ The camera was manually focused and tuned for each measurement, and resultant images were analyzed using ImageJ to determine water droplet boundaries and calculate contact angles using an ellipse best fit. Samples of PAC-1050 and AWPAC were shipped to the Particle Testing Authority (Norcross, GA) for Brunauer–Emmett–Teller (BET) surface area and micropore size analyses via a Tristar II Plus surface area and pore size analyzer, while WWPAC was analyzed using a Microtrac Belsorp Max surface area and pore size analyzer.

### 2.4 PFAS Quantification

PFAS concentrations were quantified using high-performance liquid chromatography tandem mass spectrometry (HPLC-MS/MS) (Agilent Technologies, 6410 Triple Quad LC/MS) equipped with an Agilent Poroshell EC-18 column (4.0 μm particle size, 2.1 mm × 150 mm); the instrument was operated in electrospray ionization (ESI) negative mode. The dual solvent mobile phase consisted of a variable ratio of 5 mM ammonium acetate (phase A) in LC/MS-grade water and 80/20 v/v methanol and acetonitrile (phase B), both LC/MS grade, with a constant eluent flow rate of 0.25 mL/min. Injection volume was 20 μL, and the total run time was 24.5 min. Optimal PFAS separation was achieved with a gradient of 90% A (0–2 min), 30% A (2–4 min), 2% A (4–18 min), and 100% A (18–24.5 min). Calibration standards were created ranging from 10 ppb to 2 ppm.

Higher-concentration (i.e., >2 mg/L) PFOA solutions were analyzed via HPLC-DAD (Agilent Technologies, 1100 Series) at λ = 205 nm. A Zorbax Eclipse Plus C18 (3.0 × 150 mm, 3.5-μm) column was used as the stationary phase. Standards were created ranging from 5 to 250 ppm. A sole mobile phase consisting of 50/50 (v/v) HPLC-grade acetonitrile and 25 mM Na_2_HPO_4_ in DI water adjusted to pH 2.1 with phosphoric acid was eluted at 0.6 mL/min.

### 2.5 PFOA Adsorption Isotherms

In duplicate, variable masses of a particular PAC sample were added to 50 mL polypropylene (PP) centrifuge tubes containing 250 ppm of PFOA in DI water. The PFOA solution was sonicated for 30 min to ensure adequate mixing and dissolution. The tubes were placed in an adjustable angle tube rack atop a shaker plate agitating at 250 rpm for 72 h to ensure adequate time to reach equilibrium.^45^ Preliminary experiments confirmed that this was sufficient time to reach stable PFAS concentrations. After being shaken, filtrate was recovered from the samples using a PP syringe and 0.22 μm PP filter (Fox Life Sciences), directly filtering 1 mL of sample into autosampler vials. A control sample containing no PAC was tested for PFOA concentrations before and after syringe filtration. HPLC-DAD was used to quantify PFOA removal, assuming that all removal of PFOA occurred via adsorption onto PAC, and that the filtration process removed an equal amount of PFOA for each filtration event. Although PP materials can adsorb some PFOA,^46^ the high concentration ranges employed here should make this phenomena negligible. PFAS adsorption capacity was quantified using eq 1. Data were fitted to nonlinear Langmuir and Freundlich isotherm models (eqs 2 and 3) using Microsoft Excel Solver’s Standard LSGRG Nonlinear tool via sum of squares error and sum of squares total *R*^2^ regression (eq 4).
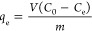
1
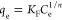
2
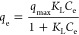
3
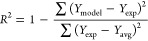
4where *q*_e_ is the mass of adsorbate adsorbed per mass of adsorbent at equilibrium (mg/g), *C*_0_ is the initial solution concentration of the adsorbate (mg/L), *C*_e_ is the equilibrium concentration of the adsorbate in solution (mg/L), *V* is the solution volume (mL), *m* is the mass of PAC added (g), *q*_max_ is the maximum adsorption capacity of adsorbate adsorbed per mass of adsorbent (mg/g), *K*_L_ is the Langmuir constant (L/mg), and *K*_F_ and 1/*n* are the Freundlich capacity and intensity parameters (mg×L^1/*n*^×g^–1^×mg^–1/*n*^ and dimensionless, respectively). *Y*_exp_*, Y*_avg_*,* and *Y*_model_ are the experimental values, average experimental value, and model values of *q*_e_, respectively (mg/g).

### 2.6 Pseudo-Second Order (PSO) Kinetic Modeling

A 500 mL beaker containing either DI water or a ∼3 mg/L synthetic NOM solution, a stir bar rotating at ∼200 rpm, 30 mg/L of a particular PAC material, and either 100 μg/L or 2 mg/L of either PFOA or PFBS were used to evaluate adsorption kinetics. At regular intervals, a 1 mL sample was pipetted from the bulk solution, filtered using a PP syringe and a 0.22 μm PP filter, and transferred to an autosampler vial for HPLC-MS/MS analysis. A filtered and unfiltered control sample was taken prior to any PAC addition, and all experiments were repeated in duplicate. It was assumed that all adsorption occurred via adsorption to the PAC or the filter material. PSO parameters were quantified using the nonlinearized PSO model, shown in eq 5, using Microsoft Excel Solver’s Standard LSGRG Nonlinear tool via sum of squares error and sum of squares total *R*^2^ regression (eq 4).
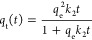
5where *q*_t_ is the mass of adsorbate adsorbed per mass of adsorbent at any time (*t*) (mg/g), *q*_e_ is the mass of adsorbate adsorbed per mass of adsorbent at equilibrium (here, assuming 4 h) (mg/g), and *k*_2_ is the pseudo-second order rate constant (L×g^–1^×min^–1^). Note, *q*_e_ calculations are the same as eq 1 assuming that equilibrium is achieved after 4 h.

### 2.7 Mixed PFAS Percent Removal vs Time

To examine the PAC material’s ability to adsorb a broader range of PFAS, we employed six PFAS in a mixed standard solution of approximately equal concentration, including PFOA, PFBS, PFNA, GenX, PFHxS, and PFOS. A beaker containing 400 mL of DI water spiked with 0.5 mg/L of each PFAS, a stir bar rotating at ∼200 rpm, and 37.5 mg/L of a particular PAC sample were used to quantify PFAS removals vs time. Periodic 1 mL samples were collected using a PP syringe equipped with a 0.22 μm PP filter and directly filtered into autosampler vials. Experiments were repeated in duplicate and were analyzed via HPLC-MS/MS. It was assumed that all PFAS removal occurred via adsorption to the PAC and to the filter membrane; control samples without PAC were taken to evaluate the effect of PFAS adsorption onto the filter, assuming this removal was equal during each sampling event.

### 2.8 Density Functional Theory (DFT) Calculations

Density functional theory (DFT) calculations were carried out within the generalized gradient approximation (GGA) using the Perdew–Burke–Ernzerhof (PBE) formulation using the Vienna Ab Initio Package (VASP) to further reveal the adsorption behavior of PFBS and PFOA on PAC surfaces with different functional groups.^47,48^ The ionic cores were described using Projected Augmented Wave (PAW) potentials, factoring in valence electrons and adopting a plane wave basis set with a kinetic energy cutoff set at 500 eV. The Gaussian smearing method was employed to permit partial occupancies of Kohn–Sham orbitals, utilizing a smearing width of 0.05 eV. Self-consistency of electronic energy was ascertained when changes in energy were below 10^–6^ eV. Geometry optimization was considered converged when force variations were under 0.02 eV/Å. Considering the complexity and uncertainty of the AC structure, a graphene-based structure was chosen as the model for quantum chemistry calculations, which consisted of 120 carbon atoms with additional −COOH, −C=O, −OH, NH_2_, or NH groups.

The adsorption energies (*E*_ads_) of adsorbate PFOA and PFBS were calculated via eq 6.
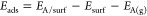
6where *E*_A/surf_, *E*_surf_, and *E*_A(g)_ are the energy of adsorbate PFBS or PFOA adsorbed on the carbonaceous surface, the energy of clean surface, and the energy of isolated PFBS or PFOA molecule in a cubic periodic box with a side length of 20 Å and 1 × 1 × 1 Monkhorst–Pack k-point grid for Brillouin zone sampling, respectively. Both protonated and deprotonated PFBS and PFOA are considered during the calculations. Two configurations for the PFBS or PFOA adsorption on the surface models were also considered to compare the effect of different adsorption directions on PFAS adsorption: (1) parallel to the adsorbed surface, and therefore, three initial adsorption systems have been fully optimized; (2) perpendicular to the surface, and an O atom near the adsorbed surface.

## 3 Results and Discussion

### 3.1 Sorbent Characterization

Comprehensive characterization techniques evidence altered chemical composition of PAC as a result of acid washing and pyrolysis treatments. Table 1 summarizes relevant PAC morphology and physicochemical parameters, including BET surface area, external surface area, micropore area and volume distribution measurements, elemental composition, and contact angle measurements.

**Table 1:** Surface Area and Micropore Data, Elemental Composition, and Contact Angle Measurements of the PAC Materials^a^

**sample parameter**	**WWPAC**	**AWPAC**	PAC-1050
BET surface area (m^2^/g)	1172.36	1266.92	1267.26
micropore volume (cm^3^/g)	0.476	0.501	0.502
micropore area (m^2^/g)	1120.23	1202.25	1205.14
external surface area (m^2^/g)	53.38	64.68	62.12
contact angle (°)	26.10	39.69	119.20
At. % oxygen	7.44	7.18	2.13
At. % carbon	92.56	92.82	97.87
PP At. % oxygen	5.48	5.06	1.59
PP At. % carbon	94.52	94.94	98.41

a Where At. % and PP At. % correspond to atomic percentage and parts per atomic percentage, respectively.

HCl treatment of the WWPAC precursor increased the BET surface area by roughly ∼100 m^2^/g and increased both the overall volume and area of available micropores, as well as the external surface area; this is likely attributed to removing residual acid-soluble impurities that are present after the PAC formation process, unblocking pores, or by producing structural changes in the carbon structure. Pyrolysis of AWPAC to produce PAC-1050 did not significantly alter the PAC, as seen by the minimal differences between the BET surface area, external surface area, micropore volume, and micropore area. This is important because available surface area can greatly affect adsorption performance metrics, with higher surface areas and greater availability of micropores, encouraging increased adsorption capacities. Furthermore, micropore size and volume also affect adsorption kinetics; since the acid wash increases these, AWPAC and PAC-1050 may have greater adsorption rates compared to that of WWPAC. All materials retain a predominantly microporous structure. As these properties are subtly different for AWPAC and PAC-1050, they can be assumed to provide negligible contributions to the observed variability in PFAS adsorption performance on PAC (see more discussion later).

Table 1 tabulizes the PAC’s elemental compositions derived via XPS; from these results, it is evident that the elemental compositions of the PAC samples change as a result of the chosen pyrolysis technique. PAC-1050 contains ∼3.5–5% lower oxygen content compared to WWPAC, resulting in less acidic and hydrophilic surfaces compared to its precursor material. HCl treatment of the PAC (AWPAC) results in a small decrease of ∼0.3–0.4% oxygen content. For the O 1S scans (Figure S1), the area counts of the peak at binding energy of 531–533 eV are roughly 3 times lower in intensity for PAC-1050 compared to that of WWPAC and AWPAC. Binding energies of 531–533 eV are associated with oxygen in hydroxyl groups or organic oxygen functionalities (e.g., carbonyl, ether, and carboxyl), and these functional groups were confirmed to be present in the materials (Tables S1 and S2).

Contact angle pictures and measurements (Figure S2) were taken to further confirm the decreased hydrophilicity of PAC-1050 that XPS suggested. As expected, WWPAC displays the greatest hydrophilic character due to its higher oxygen content and has a contact angle of 26.10°. The slight reduction in oxygen content for AWPAC can be further confirmed by its higher contact angle of 39.69°. PAC-1050 demonstrates a much greater hydrophobic character compared to the other materials, seen by its substantially higher contact angle of 119.20°. Greater hydrophobicity was also visually observed during experiments (seen in Figure S3), as PAC-1050 tended to aggregate and not to be well dispersed throughout a well-mixed aqueous matrix in comparison to WWPAC and AWPAC.

**Figure 1:**
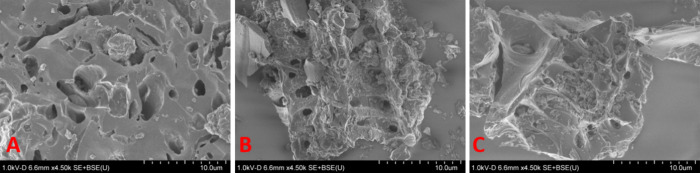
SEM images of (A) WWPAC, (B) AWPAC, and (C) PAC-1050 taken at 10 μm resolution.

Shown in Figure 1, SEM images of the PAC samples were taken to visualize the PAC surface and determine whether the treatments produced any major structural changes. From visualization, it is clear that the treatments do not produce any significant changes to the variable pore sizes and architecture of the activated carbon, retaining the original PAC morphology.

### 3.2 PFOA Adsorption Isotherms

**Figure 2:**
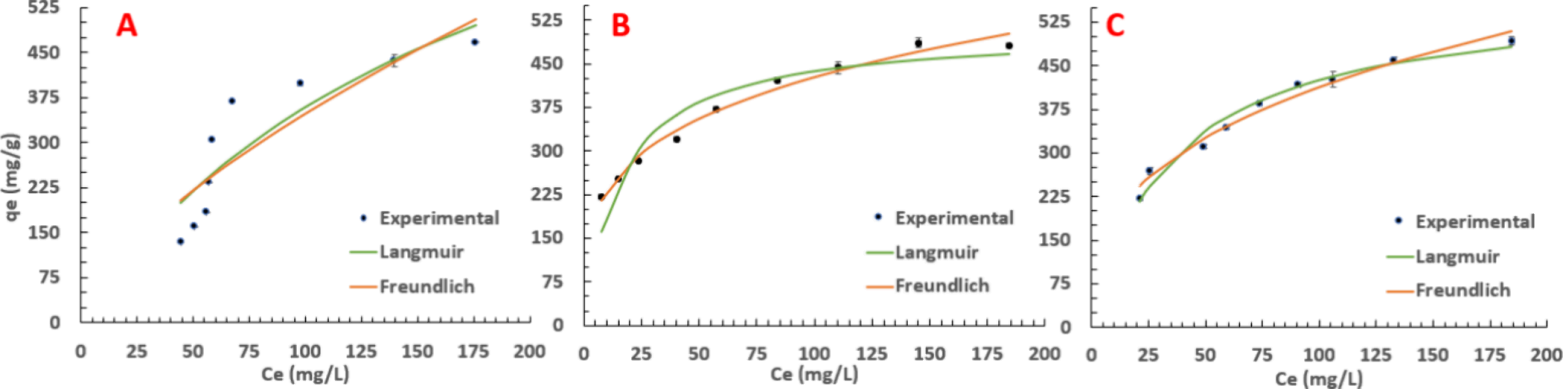
PFOA adsorption isotherms for (A) WWPAC, (B) AWPAC, and (C) PAC-1050, depicting experimental data fitted to the Freundlich and Langmuir isotherm models. Conditions: 250 mg/L PFOA in DI water and variable PAC addition.

PFOA, at a starting concentration of 250 mg/L, was chosen as the candidate PFAS to conduct single-solute batch adsorption isotherms with PAC materials. Variable masses of PAC (5–50 mg) were added in duplicate to estimate error, and all PAC types were given ample time to reach equilibrium (72 h). Figure 2 shows plots of *q*_e_ vs *C*_e_ and the respective Langmuir and Freundlich isotherm fittings, the parameters of which are tabulated in Table 2.

**Table 2:** Adsorption Isotherm Parameters for 250 mg/L PFOA Adsorption onto PAC^a^

**PAC material**	**Freundlich parameters**	**Langmuir parameters**
WWPAC	*K*_F_ = 16.92 ± 0.82	*K*_L_ = 0.00574 ± 4.2 × 10^–4^
	1/*n* = 0.657 ± 0.010	*q*_max_ = 985.04 ± 42.84
	*R*^2^ = 0.760	*R*^2^ = 0.790
AWPAC	*K*_F_ = 125.88 ± 2.17	*K*_L_ = 0.0618 ± 0.0060
	1/*n* = 0.265 ± 0.003	*q*_max_ = 505.87 ± 7.46
	*R*^2^ = 0.977	*R*^2^ = 0.873
PAC-1050	*K*_F_ = 85.99 ± 7.58	*K*_L_ = 0.0283 ± 0.0044
	1/*n* = 0.341 ± 0.020	*q*_max_ = 571.47 ± 26.20
	*R*^2^ = 0.960	*R*^2^ = 0.949

a *q*_max_ (mg/g), *K*_L_ (L/mg), *K*_F_ (mg×L_^1/*n*^_×g_^–1^_×mg_^–1/*n*^_), *n* (dimensionless).

AWPAC and PAC-1050 display L-type isotherms, which are characterized by increasing adsorption capacity values with an increasing solute concentration until the number of adsorption sites become saturated. Expectedly, there is strong affinity for the solute to PAC compared to water, and this isotherm shape suggests that adsorption occurs due to relatively strong forces (e.g., hydrophobic interactions). Interestingly, WWPAC demonstrates a sigmoidal-shaped *q*_e_ vs *C*_e_ plot, suggesting cooperative adsorption at *C*_e_ ∼50 mg/L likely occurring due to hydrophobic interactions (i.e., initial PFOA adsorption encourages more PFOA adsorption); this can be seen by a great change in *q*_e_ despite a little change in *C*_e_. The effect of HCl washing likely (A) removed residual acid-soluble impurities on the precursor material and (B) unblocked some pores on the surface of the carbon, facilitating higher PFOA adsorption at lower *C*_e_ values for both AWPAC and PAC-1050, while WWPAC plateaus at a *q*_e_ value of ∼450 mg/g, AWPAC and PAC-1050 plateau at a *q*_e_ value of ∼470 mg/g, suggesting that overall adsorption capacity at equilibrium is limited by the adsorbent’s total surface area since there was an insubstantial increase to overall BET surface area. Both AWPAC and PAC-1050 demonstrate very similar adsorption isotherm behavior, adhering well to both of the tested adsorption models. Expectedly, the Freundlich model is a better fitting model than the Langmuir model, which is typical of AC-based adsorbents due to heterogeneous surfaces and oftentimes multilayer adsorption mechanisms.

### 3.3 Kinetics of PFOA and PFBS Adsorption onto PAC Materials

**Figure 3:**
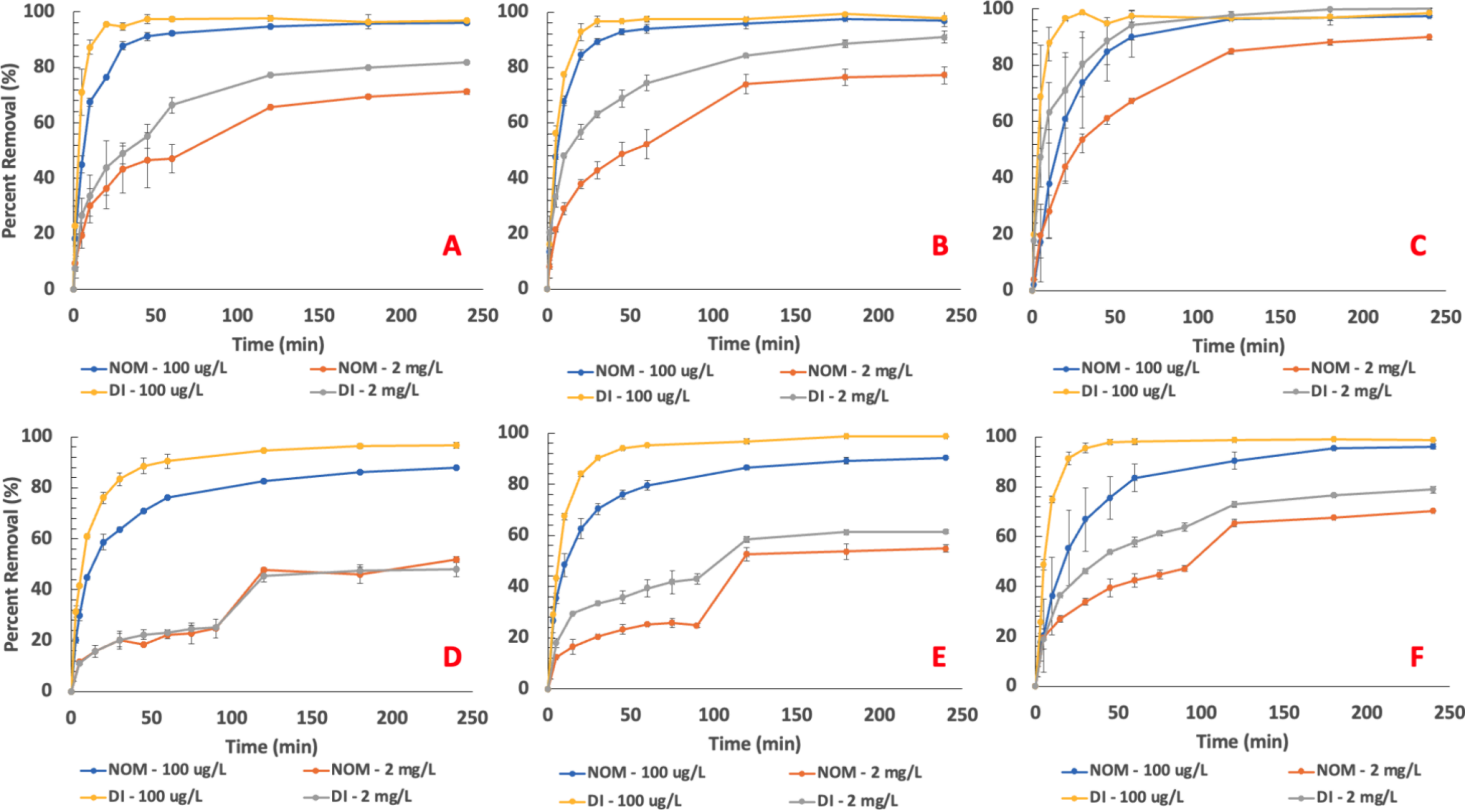
Percent removal vs time for (A) WWPAC, PFOA (B) AWPAC, PFOA, (C) PAC-1050, PFOA (D) WWPAC, PFBS, (E) AWPAC, PFBS, and (F) PAC-1050, PFBS. Conditions: 30 mg/L PAC, 3 mg/L NOM, 100 μg/L or 2 mg/L PFAS concentration.

PFOA or PFBS, two PFAS of different chain lengths and functional head groups, were chosen as model PFAS to evaluate the kinetics of adsorption onto the different PAC materials. Figure 3 shows percent removal vs time for each of the PACs, comparing the removal of PFOA and PFBS in both DI and synthetic NOM solutions, at PFAS concentrations of either 100 μg/L or 2 mg/L. PFAS were tested separately, and each experiment was repeated in duplicate.

It is clear that PAC-1050 outperforms both AWPAC and WWPAC in terms of the removal rate for both PFAS molecules and in both aqueous matrices at higher concentrations of 2 mg/L PFAS; further, PAC-1050 achieves a higher percent removal value for all test conditions after 4 h compared to the other PAC types at this concentration. At 100 μg/L PFOA, there is a little difference in the removal rate for all three PAC types in either matrix, suggesting that the molecule is easily removed at this concentration level; however, for PFBS, PAC-1050 slightly outperforms in the DI water matrix in terms of the removal rate and, for the NOM case, in the overall removal value. Near complete PFAS removal at lower concentrations is expectedly much easier, aligned with the high adsorption capacity and high surface area of the PAC. AWPAC outperforms WWPAC for both PFOA and PFBS adsorption, especially in DI water; however, this effect is less pronounced in the presence of NOM, suggesting that the hydrophilic portions of NOM retard AWPAC’s adsorption rate more so compared to that of PAC-1050. PFBS expectedly has a lower overall percent removal value and rate of adsorption compared to PFOA, which can be attributed to its smaller molecular size and smaller intramolecular adsorptive forces. These results suggest that PAC-1050 has a higher affinity for PFAS molecules over both water and NOM compared with the other PACs.

**Figure 4:**
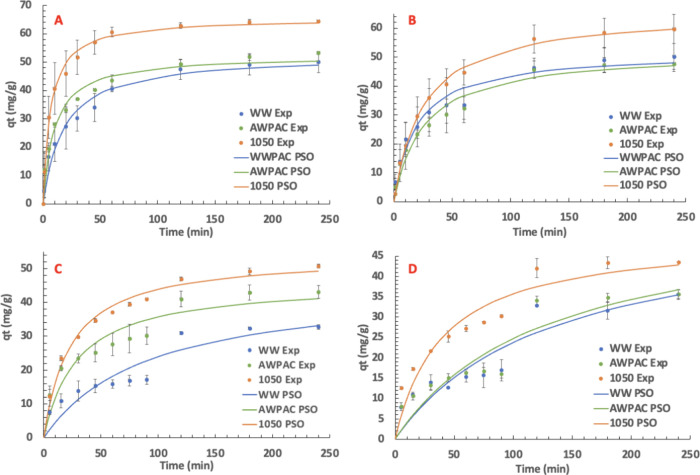
Pseudo-second-order (PSO) kinetic modeling for (A) PFOA in DI water, (B) PFOA in NOM, (C) PFBS in DI water, and (D) PFBS in NOM. Conditions: 2 mg/L individual PFAS, 30 mg/L PAC, 3 mg/L NOM.

The nonlinear PSO model was chosen to evaluate kinetics for the adsorption for 2 mg/L PFOA and PFBS onto 30 mg/L of the various PACs. Previous works have shown that the PSO model is effective in examining the kinetics of several different PFAS adsorption onto carbonaceous materials.^49,50^Figure 4 and Table 3 show the PSO model results over the experimental data for each test and the tabulated PSO parameters, respectively. It is clear that the PSO model more accurately fits the PFOA data, with the lowest coefficient of determination of 0.962. In contrast, the lowest coefficient of determination for PFBS is 0.843, observed in NOM solution. This could be explained due to PFBS’s lower interactive intramolecular forces with PAC and its smaller molecular size, which is discussed in more detail in Section 3.5. Still, the PSO model can reasonably predict the PFBS adsorption rate.

**Table 3:** Pseudo-Second Order (PSO) Kinetic Parameters

	**WWPAC**		**AWPAC**		PAC-1050	
parameter	**DI**	**NOM**	**DI**	**NOM**	**DI**	**NOM**
**PFOA**						
*q*_e, 4 h_ (mg/g)	52.91 ± 0.16	53.61 ± 0.54	52.45 ± 1.93	52.08 ± 3.75	66.47 ± 0.69	66.10 ± 1.92
*k*_2_ (L×g^–1^×min^–1^)	1.1 ± 0.8 × 10^–3^	1.1 ± 0.9 × 10^–3^	2.0 ± 0.1 × 10^–3^	7.6 ± 0.4 × 10^–4^	2.4 ± 1.0 × 10^–4^	6.0 ± 3.0 × 10^–4^
*R*^2^	0.976	0.967	0.962	0.967	0.992	0.991
**PFBS**						
*q*_e, 4 h_ (mg/g)	47.22 ± 12.56	65.39 ± 0.01	46.30 ± 2.48	41.73 ± 2.28	53.92 ± 0.33	40.45 ± 3.88
*k*_2_ (L×g^–1^×min^–1^)	3.1 ± 2.7 × 10^–4^	8.5 ± 0.8 × 10^–5^	7.3 ± 1.1 × 10^–4^	1.4 ± 0.2 × 10^–4^	8.0 ± 0.9 × 10^–4^	2.9 ± 1.5 × 10^–3^
*R*^2^	0.881	0.843	0.911	0.835	0.984	0.874

### 3.4 Removal of Mixed PFAS

**Figure 5:**
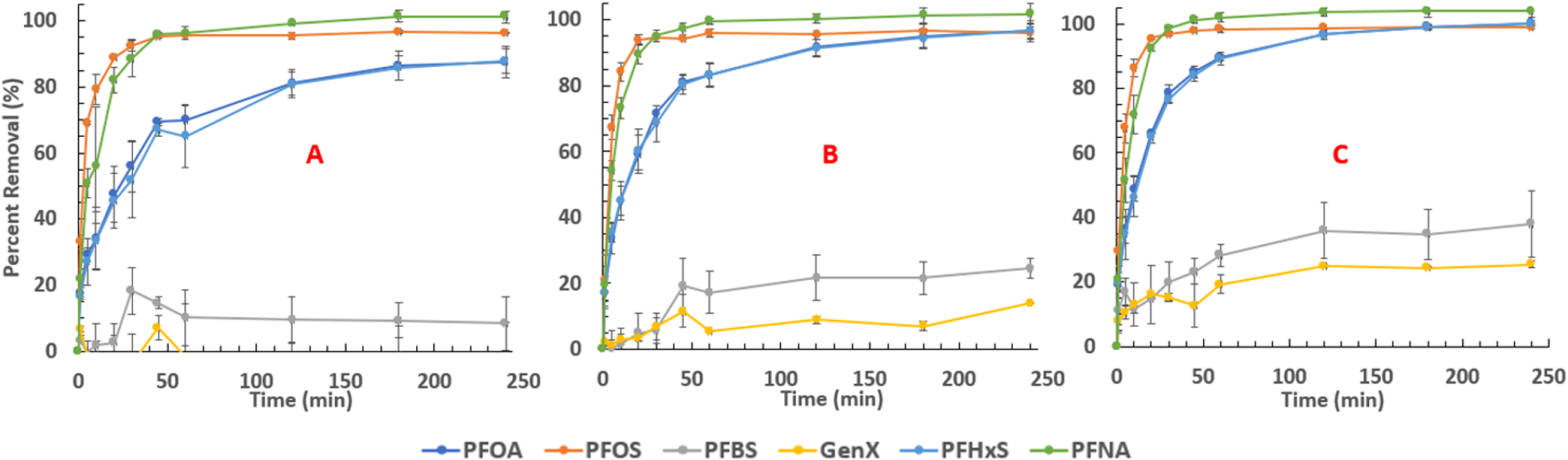
Mixed PFAS percent removal versus time for (A) WWPAC, (B) AWPAC, and (C) PAC-1050. Conditions: 500 μg/L of each PFAS, 37.5 mg/L PAC in DI water.

A solution containing approximately 500 μg/L each of PFOA, PFBS, GenX, PFHxS, PFNA, and PFOS was used to evaluate the PAC materials’ adsorption efficiency in a competitive system. Figure 5 shows the percent removal vs time plots for each PAC type. For perfluorosulfonic acids (PFSAs) and perfluorocarboxylic acids (PFCAs), the carbon chain length directly relates to the compound’s hydrophobicity, with longer-chain molecules possessing greater hydrophobicity than that of their shorter-chain counterparts. As such, this explains the trend of more readily removed PFAS (i.e., PFOS > PFHxS > PFBS and PFNA > PFOA). GenX, possessing an ether moiety, is the least readily removed PFAS for all PAC types, likely due to its higher hydrophilicity. PAC-1050 outperforms both WWPAC and AWPAC in that it achieves both faster and higher removal rates of all PFAS, notably PFOA and PFHxS, and removes the greatest amount of both PFBS and GenX over a span of 4 h. The observed adsorption/desorption, indicated by increasing then decreasing amounts of percent removal for PFBS and GenX, can be attributed to the PAC initially adsorbing some of the less favorable PFAS and then desorbing them for larger and more hydrophobic PFAS as equilibrium is approached.

### 3.5 Density Functional Theory Calculations

DFT calculations were performed to quantify the adsorption energies of either PFOA or PFBS adsorption onto a graphene-based carbon skeleton with different theoretical oxygen- and nitrogen-containing surface group moieties: C=O, −COOH, −OH, −NH, and −NH_2_. Specifically, one material containing 5% oxygen content (containing all test oxygenated functional groups) was compared with a variety of other test candidates containing 2% oxygen content, with or without the aforementioned functional groups. It is well-known that the hydrophilic heads of PFAS possess negative electrostatic potentials.^51^ After comparing two different configurations of PFAS adsorption onto the carbon surface, it was observed that their hydrophilic heads align more closely to the tested functional groups once their geometry is fully optimized. Both PFOA and PFBS are strongly attracted to the N-containing groups due to the positive electrostatic potential of these functional groups. Table 4 tabulates the resulting adsorption energies of either neutral or anionic PFOA or PFBS onto these carbon structures with and without these various groups at variable oxygen concentrations.

**Table 4:** DFT Calculation Results for the Adsorption Energies (in eV) of Neutral and Anionic Forms of PFOA and PFBS and Water onto Graphene Structures with Various Functional Groups

test condition	PFBS	PFOA	anionic PFBS	anionic PFOA	H_2_O
PAC with C=O, −COOH, and −OH (5% oxygen)	–0.13013	–0.1935	–1.2012	–1.5013	–1.6532
PAC with COOH (2% oxygen)	–0.2122	–0.3651	–1.4543	–1.6798	–0.4321
PAC with C=O (2% oxygen)	–0.23673	–0.26543	–1.3211	–1.5244	–0.38854
PAC with C–OH (2% oxygen)	–0.3435	–0.4026	–1.4843	–1.6732	–0.35858
PAC with COOH and NH2 (2% oxygen, 0.5%N)	–0.53232	–0.58244	–1.6211	–1.8912	–0.82123
PAC with C=O and NH2 (2% oxygen, 0.5%N)	–0.36854	–0.4269	–1.4343	–1.7456	–0.83122
PAC with C–OH and NH2 (2% oxygen, 0.5%N)	–0.49763	–0.51749	–1.5621	–1.8134	–0.82654
PAC with COOH and N (2% oxygen, 0.5%N)	–0.58432	–0.61224	–1.6234	–1.9055	–0.92321
PAC with C=O and N (2% oxygen, 0.5%N)	–0.39462	–0.41237	–1.4743	–1.8122	–0.90356
PAC with C–OH and N (2% oxygen, 0.5%N)	–0.52931	–0.54231	–1.5839	–1.8542	–0.88834

Corresponding to the DFT results pertaining to oxygenated carbon structures, the −OH group-modified carbonaceous surfaces had the largest adsorption energies for PFBS and PFOA, with values of −0.34 and −0.40 eV, respectively, outperforming both −COOH and C=O groups in terms of adsorption energies for both PFAS. While PFBS has a little difference in adsorption energy for PAC containing either −COOH or C=O, PFOA more favorably adsorbs onto PAC(−COOH) than PAC(C=O). These differences can be explained by organic functional group interactions. The −OH group is a strong hydrogen donor, whereas anionic PFAS are strong hydrogen acceptors. The −COOH group, which is generally negatively charged in water matrices due to low p*K*_a_ values of 3–5, becomes increasingly ionized at higher pH values, reducing possibilities of favorable interaction due to electrostatic repulsions with PFAS heads. While −COOH can still interact via hydrogen bonding or ionic interactions, these interactions are generally weaker than the −OH group. The C=O group, while able to participate in hydrogen bonding, is a hydrogen acceptor similarly as PFAS, hence providing weaker interaction with PFAS than that of −OH or −COOH. These results suggest that both the O-containing functional group type and overall oxygen percentage are both important factors in the adsorption process of PFAS onto PAC. In all cases, the adsorption energies of PFBS were significantly lower than those of PFOA, which is likely due to the lower electronegativity of the hydrophilic head of PFBS compared to PFOA,^52^ as well as its smaller molecular size. Hydrogen bonding between the deprotonated anionic PFAS species and functional groups is the main mechanism, and it is unlikely that environmental water matrices will contain protonated PFAS due to their low p*K*_a_ values. The deprotonated PFAS or anionic forms of PFAS adsorb more strongly than that for the protonated neutral form, as indicated by the higher negative *E*_ads_ seen in Table 4.

Besides electrostatic interactions and hydrogen bonding, hydrophobic interactions are also of great importance in the adsorption of PFAS onto carbon surfaces. Li et al. explored oxygen contents of multiwalled nanotubes (MWNTs) for PFOA and PFOS adsorption capacity and found that adsorption capacity decreases dramatically with the increasing of oxygen contents due to reduced hydrophobic interaction.^53^ Interestingly, the test candidate with 5% oxygen content has the highest adsorption energies for both PFOA and PFOS out of all of the test candidates, confirming that reducing the oxygen content and increasing hydrophobicity can enhance adsorption favorability. Thus, hydrophobicity is of greater importance than electrostatic interactions or hydrogen bonding. As shown in Table 4, the adsorption energy of H_2_O onto PAC dramatically increased after a decrease in oxygen content, which suggests more favorable adsorption for both PFOA and PFBS onto PAC, with weaker H_2_O competition for the surface sites. Since the hydrophobicity of PFOA is higher than that of PFBS, this explains its more favorable adsorption onto PAC in all test cases.

The adsorption of PFAS onto PAC for the 2% amino or pyrrolic functionalized surface was significantly better than that of only oxygen-containing functional groups, suggesting that hydrophobic interactions are not the only interactions governing this adsorption process. Moreover, with either amino groups or pyrrolic N incorporated into carbonaceous surfaces, the adsorption energies of PFBS and PFOA both significantly increase due to the positive charge of the nitrogen group and the enhancement of hydrogen bonding to PFAS molecules. Although no nitrogenated PAC materials were experimentally tested here, the incorporation of nitrogenated moieties seems to suggest improved PFAS adsorption performance onto PAC. This improvement is likely attributed to enhanced basicity and electrostatic interactions. These results suggest that this is a viable research pathway to explore, although the syntheses of such materials should take care to not drastically reduce a sorbent’s overall surface area and adsorption capacity, which could result in decreased performance.

## Conclusions and Environmental Implications

This work sheds further light on the adsorption of PFAS onto carbonaceous adsorbents, particularly highlighting that enhanced PFAS adsorption kinetics can be achieved by enhancing the hydrophobicity of the sorbent. Since AC is typically produced using high-temperature processes in the presence of steam, the fabrication process could be possibly altered to favor a more hydrophobic activated carbon. Ways to reduce oxygen content during AC synthesis might include selecting for precursor materials with lower oxygen content, precisely controlling the carbonization conditions and maintaining inert atmospheres to prevent reoxygenation, using reducing agents during chemical activation, or, like in this work, employing posttreatment strategies like pyrolysis.

PAC is generally utilized in DWT process trains periodically, especially when utilities may have difficulties adhering to permit regulations due to unexpected changes in source water (e.g., stormwater turbidity events). Should DWT utilities be pushed by either more stringent concentration regulations or public fervor to more chemically independent and benign “Minus” approaches in the future, PAC will become an increasingly relevant tool in the water treatment toolbox.

Notably, the procedures used to prepare the PAC materials utilized here have not been optimized, and many variables might impact the resultant PAC’s composition (e.g., pyrolysis time and temperature, selection of raw material for AC synthesis). Therefore, further exploration of this phenomena is warranted to determine whether performance metrics can be enhanced further. Future studies should also examine the adsorption of less common PFAS of novel concern (e.g., fluorotelomers, perfluoroalkyl phosphonic acids (PFPAs)) onto PAC and other novel adsorbents, as well as investigating competitive effects from other cocontaminants in complex mixtures containing PFAS. For instance, hydrophobic, non-PFAS compounds such as hydrocarbons are commonly found at sites impacted by aqueous film forming foams (AFFFs), in addition to PFAS.

As our DFT calculations suggest, nitrogenating PAC materials with −NH or −NH_2_ moieties could also be a favorable route for enhancing the free energies of PFAS adsorption, although any synthesis pathway should take care to maintain (or, even better, to bolster) a precursor material’s surface area. Despite our speculation, the results presented here add further knowledge of PFAS adsorption onto PAC and confirm the importance of hydrophobicity during this process.

## Supporting Information Available

C 1S and O 1S XPS scans for PAC-1050, AWPAC, and WWPAC (Figure S1); XPS C 1S scan data for PAC-1050, AWPAC, and WWPAC (Table S1); XPS O 1S scan data for PAC-1050, AWPAC, and WWPAC (Table S2); contact angle pictures and measurements for PAC-1050, AWPAC, and WWPAC (Figure S2); and photograph of experimental setup to support increased hydrophobicity for PAC-1050 (Figure S3) (PDF)
