# Supplementary material for: Improving the Hydrophobicity of Powder Activated Carbon to Enhance the Adsorption Kinetics of Per- and Polyfluoroalkyl Substances
Source: ACS ES T Water. 2025 Apr 7;5(5):2322–32. doi: 10.1021/acsestwater.4c01222 (PMC12070405; doi:10.1021/acsestwater.4c01222)
Supplement: Supplementary file 1 — ew4c01222_si_001.pdf [file ew4c01222_si_001.pdf]

Supporting Information

Improving the Hydrophobicity of Powder Activated Carbon to Enhance the Adsorption Kinetics of Per- and Polyfluoroalkyl Substances

Elliot Reid,[1] Qingquan Ma,[1] Lan Gan,[1] Jiahao He,[1] Thomas Igou,[1] Ching-Hua Huang,[1] and Yongsheng Chen[1,*]

[1] School of Civil and Environmental Engineering, Georgia Institute of Technology, Atlanta, Georgia, 30332, United States

[*]**Corresponding Author**: yongsheng.chen@ce.gatech.edu

Number of Figures – 3

Number of Tables – 2

Number of Pages - 5

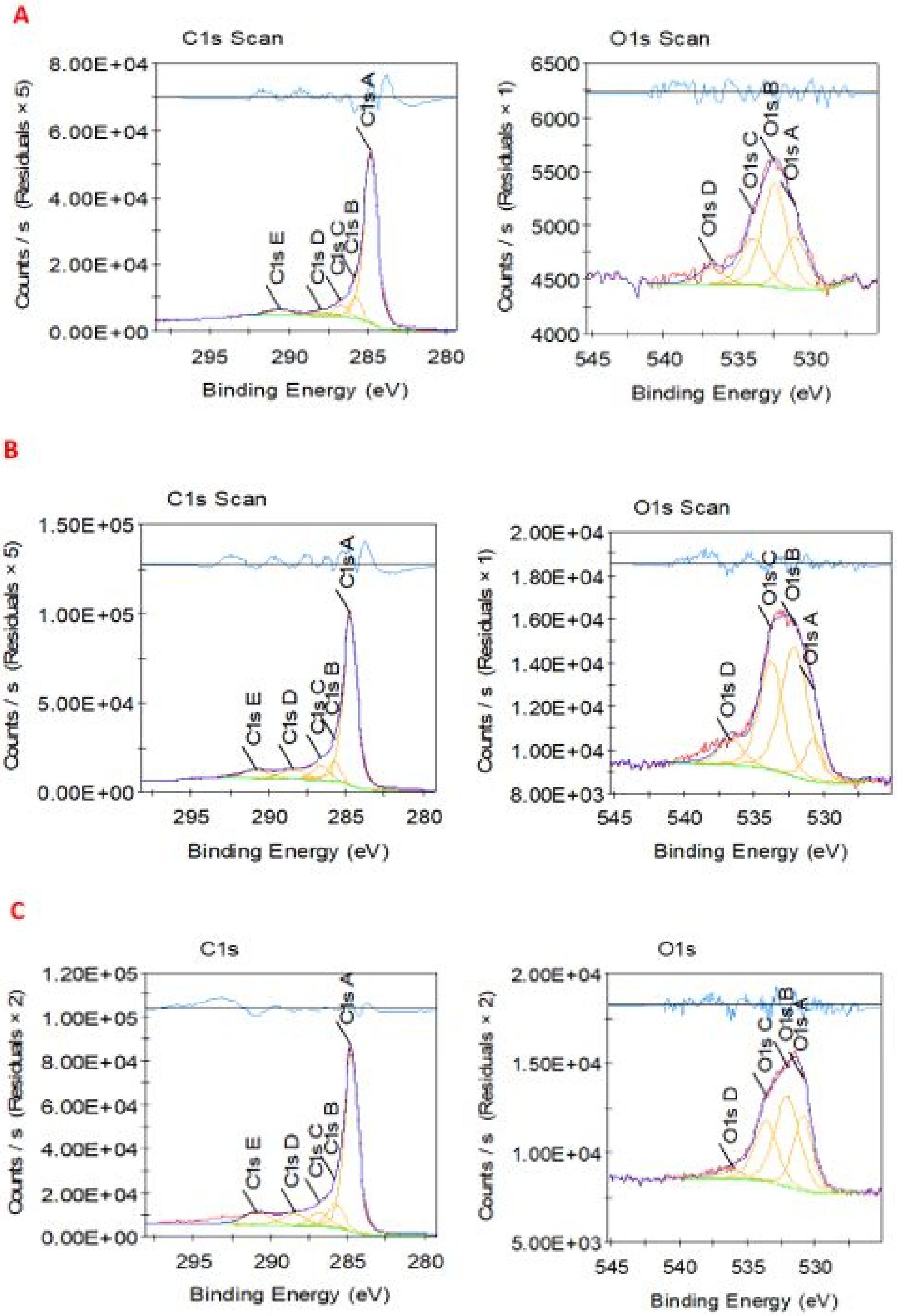

Figure S1 – C 1S and O 1S scans derived from x-ray photoelectron spectroscopy (XPS) for (A) PAC-1050, (B) AWPAC, and (C) WWPAC

Table S1 – C 1S scan data information from XPS

C 1s Scan Information			
**PAC-1050**			
**Peak**	**Binding Energy (eV)**	**Peak Assignment**	**%**
A	284.8	C-C/C-H, graphitic carbon	78.12
B	285.8	C-O-, alcoholic or etheric carbon	8.59
C	286.7	C=O, carbonyl carbon	5.47
D	287.9	COO, carboxyl or ester	4.69
E	290.5	π-π* transition	3.13
**AWPAC**			
**Peak**	**Binding Energy (eV)**	**Peak Assignment**	**%**
A	284.7	C-C/C-H, graphitic carbon	71.43
B	285.8	C-O-, alcoholic or etheric carbon	7.14
C	286.6	C=O, carbonyl carbon	7.86
D	288.4	COO, carboxyl or ester	7.14
E	290.7	π-π* transition	6.43
**WWPAC**			
**Peak**	**Binding Energy (eV)**	**Peak Assignment**	**%**
A	284.8	C-C/C-H, graphitic carbon	65.36
B	285.8	C-O-, alcoholic or etheric carbon	10.46
C	286.8	C=O, carbonyl carbon	7.19
D	288.5	COO, carboxyl or ester	7.84
E	290.9	π-π* transition	9.15

Table S2 – O 1S scan data information from XPS

O 1s Scan Information			
**PAC-1050**			
**Peak**	**Binding Energy (eV)**	**Peak Assignment**	**%**
A	531.0	C=O, carbonyl	47.84
B	532.4	C-OH	21.53
C	533.9	C-O, oxygen singly bonded to carbon	22.97
D	536.7	Physiosorbed CO_2 or water	7.66
**AWPAC**			
**Peak**	**Binding Energy (eV)**	**Peak Assignment**	**%**
A	530.7	C=O, carbonyl	10.18
B	532.1	C-OH	46.30
C	533.8	C-O, oxygen singly bonded to carbon	34.72
D	536.6	Physiosorbed CO_2 or water	8.80
**WWPAC**			
**Peak**	**Binding Energy (eV)**	**Peak Assignment**	**%**
A	531.3	C-O, oxygen singly bonded to carbon	41.84
B	533.0	C-OH	26.78
C	534.3	C=O, carbonyl	27.20
D	536.8	Physiosorbed CO_2 or water	4.18

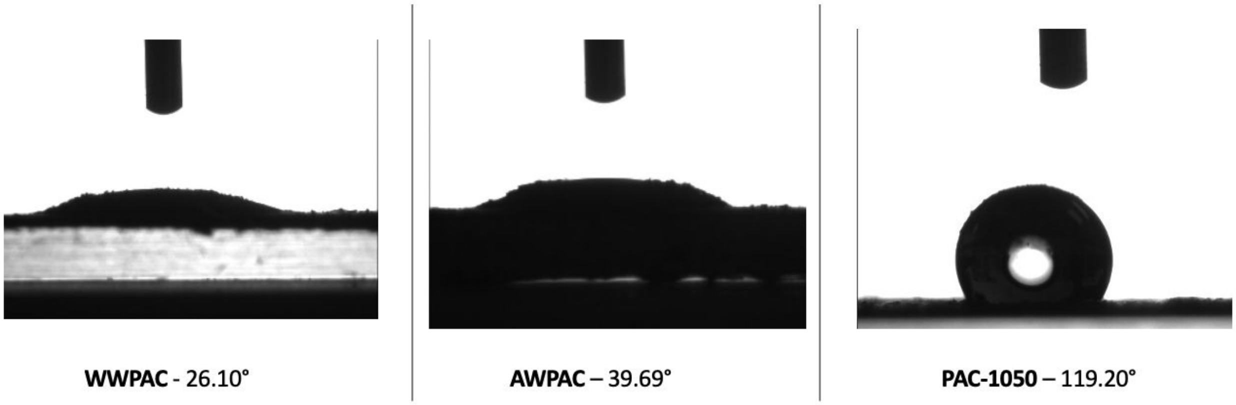

Figure S2 – Contact angle pictures and measurements of the PAC samples

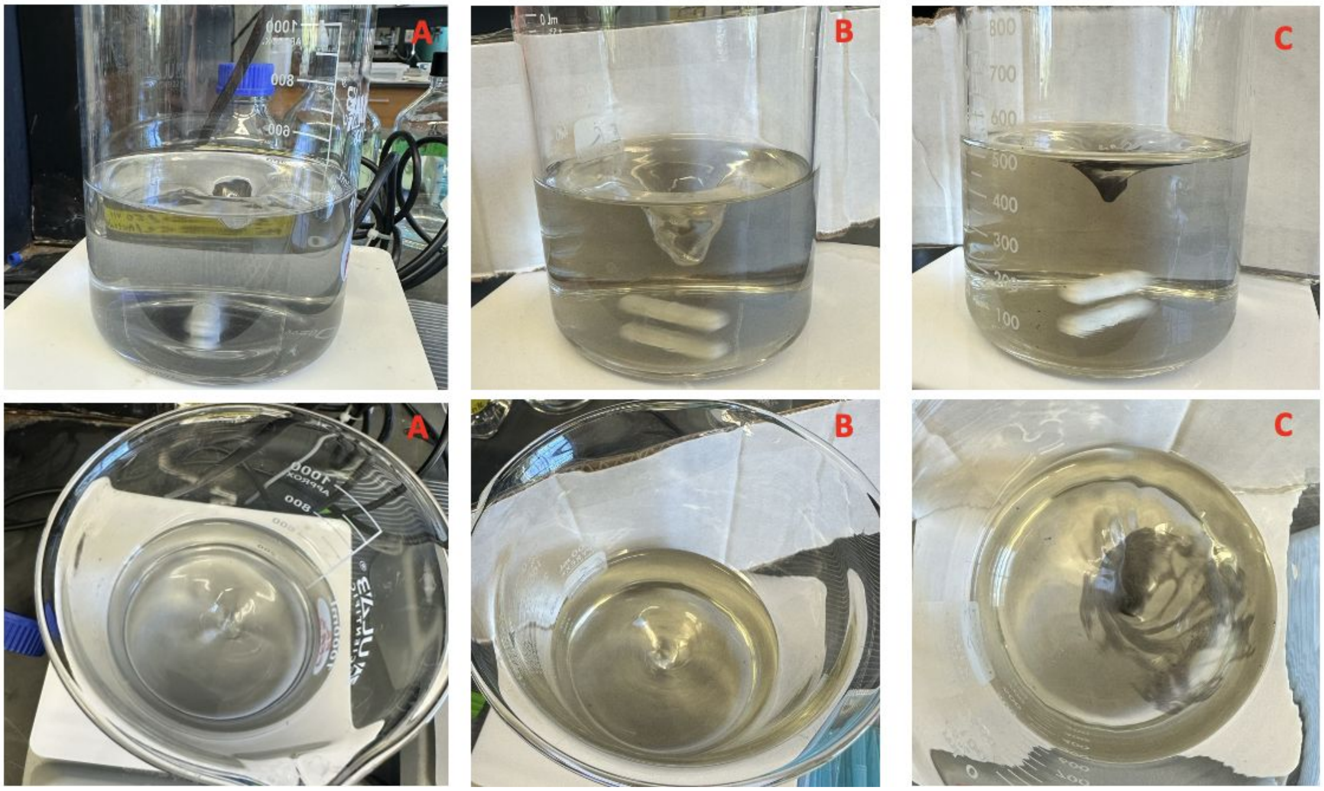

Figure S3 – Side and top view of (A) WWPAC, (B) AWPAC, and (C) PAC-1050 during well-mixed kinetic experiments
